# Supplementary material for: Deep context-attentive transformer transfer learning for financial forecasting
Source: PeerJ Comput Sci. 2025 Jun 30;11:e2983. doi: 10.7717/peerj-cs.2983 (PMC12453852; doi:10.7717/peerj-cs.2983)
Supplement: Supplemental Information 1 [file peerj-cs-11-2983-s001.docx]

Table S1. Statistical significance and confidence interval analysis.

| **Comparison** | **Metric** | **p-value** | **Significant?** | **Confidence interval** |
| --- | --- | --- | --- | --- |
| vs CNN-BiLSTM-Attention | MSE | 0.0156 | Yes (p < 0.05) | (0.0418, 4.6997) |
| vs CNN-BiLSTM-Attention | MAE | 0.0156 | Yes (p < 0.05) | (0.0835, 1.3958) |
| vs CNN-BiLSTM-Attention | R² | 0.0156 | Yes (p < 0.05) | (0.2014, 2.1903) |
| vs CNN-GRU-Attention | MSE | 0.0156 | Yes (p < 0.05) | (0.0169, 0.7762) |
| vs CNN-GRU-Attention | MAE | 0.0156 | Yes (p < 0.05) | (0.0352, 0.4728) |
| vs CNN-GRU-Attention | R² | 0.0156 | Yes (p < 0.05) | (0.0481, 0.3596) |
| vs Deep-Transformer | MSE | 0.0156 | Yes (p < 0.05) | (0.0121, 3.6620) |
| vs Deep-Transformer | MAE | 0.0156 | Yes (p < 0.05) | (0.0188, 1.3161) |
| vs Deep-Transformer | R² | 0.0156 | Yes (p < 0.05) | (0.0745, 3.3245) |
| vs Informer | MSE | 0.0156 | Yes (p < 0.05) | (0.0150, 6.7249) |
| vs Informer | MAE | 0.0156 | Yes (p < 0.05) | (0.0261, 2.0119) |
| vs Informer | R² | 0.0156 | Yes (p < 0.05) | (0.0754, 6.2657) |
| vs CNN-BiLSTM-Attention (TL) | MSE | 0.0313 | Yes (p < 0.05) | (0.0385, 6.0345) |
| vs CNN-BiLSTM-Attention (TL) | MAE | 0.0313 | Yes (p < 0.05) | (0.0851, 1.6188) |
| vs CNN-BiLSTM-Attention (TL) | R² | 0.0313 | Yes (p < 0.05) | (0.2252, 2.8186) |
| vs CNN-GRU-Attention (TL) | MSE | 0.0313 | Yes (p < 0.05) | (0.0158, 0.7087) |
| vs CNN-GRU-Attention (TL) | MAE | 0.0313 | Yes (p < 0.05) | (0.0353, 0.4648) |
| vs CNN-GRU-Attention (TL) | R² | 0.0313 | Yes (p < 0.05) | (0.0740, 0.3275) |
| vs Deep-Transformer (TL) | MSE | 0.0313 | Yes (p < 0.05) | (0.0141, 2.3963) |
| vs Deep-Transformer (TL) | MAE | 0.0313 | Yes (p < 0.05) | (0.0240, 0.8957) |
| vs Deep-Transformer (TL) | R² | 0.0313 | Yes (p < 0.05) | (0.1071, 2.1689) |
| vs Informer (TL) | MSE | 0.0313 | Yes (p < 0.05) | (0.0158, 7.4440) |
| vs Informer (TL) | MAE | 0.0313 | Yes (p < 0.05) | (0.0252, 2.1503) |
| vs Informer (TL) | R² | 0.0313 | Yes (p < 0.05) | (0.0933, 6.9196) |
