# Supplementary material for: Deep context-attentive transformer transfer learning for financial forecasting
Source: PeerJ Comput Sci. 2025 Jun 30;11:e2983. doi: 10.7717/peerj-cs.2983 (PMC12453852; doi:10.7717/peerj-cs.2983)
Supplement: Supplemental Information 2 [file peerj-cs-11-2983-s002.docx]

Table S2. Comparison of using and not using rotary positional encoding.

|  | **(2CAT not using**  **rotary positional encoding**  **– proposed technique)** | | | **(2CAT using**  **rotary positional encoding**  **– ablation study)** | | |
| --- | --- | --- | --- | --- | --- | --- |
|  | **MSE** | **MAE** | **R²** | **MSE** | **MAE** | **R²** |
| DJIA | 0.0655 | 0.2023 | 0.9169 | **0.0598** | **0.1904** | **0.9241** |
| N225 | 0.3081 | 0.4242 | 0.7129 | **0.2979** | **0.4155** | **0.7224** |
| HSI | 0.0146 | 0.0945 | 0.8212 | **0.0138** | **0.0925** | **0.8306** |
| SSE | 0.0457 | 0.1626 | 0.8782 | **0.0431** | **0.1573** | **0.8850** |
| BSE | 0.0467 | 0.1692 | 0.8881 | **0.0437** | **0.1596** | **0.8954** |
| SET | 0.1606 | 0.3189 | 0.9094 | **0.1562** | **0.3171** | **0.9119** |
