# Supplementary material for: Deep context-attentive transformer transfer learning for financial forecasting
Source: PeerJ Comput Sci. 2025 Jun 30;11:e2983. doi: 10.7717/peerj-cs.2983 (PMC12453852; doi:10.7717/peerj-cs.2983)
Supplement: Supplemental Information 3 [file peerj-cs-11-2983-s003.docx]

Table S3. Comparison of using and not using rotary positional encoding in TL.

|  | **Transfer Learning**  **(2CAT not using**  **rotary positional encoding**  **– proposed technique)** | | | **Transfer Learning**  **(2CAT using**  **rotary positional encoding**  **– ablation study)** | | |
| --- | --- | --- | --- | --- | --- | --- |
|  | **MSE** | **MAE** | **R²** | **MSE** | **MAE** | **R²** |
| N225 | 0.3035 | 0.4242 | 0.7172 | **0.2889** | **0.4138** | **0.7308** |
| HSI | 0.0137 | 0.0933 | 0.8321 | **0.0131** | **0.0901** | **0.8396** |
| SSE | 0.0452 | 0.1641 | 0.8796 | **0.0430** | **0.1569** | **0.8854** |
| BSE | 0.0428 | 0.1604 | 0.8974 | **0.0428** | **0.1603** | **0.8975** |
| SET | 0.1566 | 0.3134 | 0.9117 | **0.1534** | **0.3101** | **0.9135** |
