# Supplementary material for: Deep context-attentive transformer transfer learning for financial forecasting
Source: PeerJ Comput Sci. 2025 Jun 30;11:e2983. doi: 10.7717/peerj-cs.2983 (PMC12453852; doi:10.7717/peerj-cs.2983)
Supplement: Supplemental Information 4 [file peerj-cs-11-2983-s004.docx]

Table S4. Comparison of proposed model results on 1-day, 3-day, and 5-day predictions.

|  | Proposed Method | | | | | | | | |
| --- | --- | --- | --- | --- | --- | --- | --- | --- | --- |
|  | 1-day prediction | | | 3-day prediction | | | 5-day prediction | | |
|  | MSE | MAE | R² | MSE | MAE | R² | MSE | MAE | R² |
| DJIA | 0.0188 | 0.1091 | 0.9771 | 0.0441 | 0.1643 | 0.9451 | 0.0655 | 0.2023 | 0.9169 |
| N225 | 0.1004 | 0.2546 | 0.9126 | 0.2097 | 0.3558 | 0.8112 | 0.3081 | 0.4242 | 0.7129 |
| HSI | 0.0059 | 0.0613 | 0.9291 | 0.0104 | 0.0809 | 0.8733 | 0.0146 | 0.0945 | 0.8212 |
| SSE | 0.0185 | 0.1044 | 0.9525 | 0.0331 | 0.1347 | 0.9130 | 0.0457 | 0.1626 | 0.8782 |
| BSE | 0.0141 | 0.0927 | 0.9676 | 0.0300 | 0.1354 | 0.9296 | 0.0467 | 0.1692 | 0.8881 |
| SET | 0.0531 | 0.1870 | 0.9700 | 0.1016 | 0.2530 | 0.9428 | 0.1606 | 0.3189 | 0.9094 |
