# Supplementary material for: Deep context-attentive transformer transfer learning for financial forecasting
Source: PeerJ Comput Sci. 2025 Jun 30;11:e2983. doi: 10.7717/peerj-cs.2983 (PMC12453852; doi:10.7717/peerj-cs.2983)
Supplement: Supplemental Information 5 [file peerj-cs-11-2983-s005.docx]

The preliminary study examined the correlation between macroeconomic indicators—such as the Consumer Price Index (CPI)—and financial indices, as shown in Table S5. The macroeconomic indicators and financial index used in this study are available on the [Statistics South Africa website](https://www.statssa.gov.za/?page_id=1847) and [South African Stock Market Index (SA40)](https://www.kaggle.com/datasets/neilslab/south-africa-stock-market-data).

**Table S5. Correlation results of different datasets.**

| **Correlation Matrix** | | **South African Indices** | | | | | **Global Indices** | |
| --- | --- | --- | --- | --- | --- | --- | --- | --- |
|  |  | **CPI1** | **CPI 2** | **CPI3** | **CPI4** | **SA40** | **DJIA** | **N225** |
| **South African Indices** | **CPI1** | 1.000 | -0.961 | -0.845 | -0.437 | 0.903 | -0.599 | -0.751 |
|  | **CPI2** | -0.961 | 1.000 | 0.859 | 0.398 | -0.899 | 0.719 | 0.682 |
|  | **CPI3** | -0.845 | 0.859 | 1.000 | 0.647 | -0.816 | 0.597 | 0.395 |
|  | **CPI4** | -0.437 | 0.398 | 0.647 | 1.000 | -0.501 | 0.368 | -0.002 |
|  | **SA40** | 0.903 | -0.899 | -0.816 | -0.501 | 1.000 | -0.624 | -0.623 |
| **Global Indices** | **DJIA** | -0.599 | 0.719 | 0.597 | 0.368 | -0.624 | 1.000 | 0.208 |
|  | **N225** | -0.751 | 0.682 | 0.395 | -0.002 | -0.623 | 0.208 | 1.000 |
| CPI1 - CPI of Information Processing CPI2 - CPI of Financial Services  CPI3 - CPI of Rice CPI4 - CPI of Tourist Income  SA40 – South African Top 40 Index | | | | | | | | |
