# Supplementary material for: Deep context-attentive transformer transfer learning for financial forecasting
Source: PeerJ Comput Sci. 2025 Jun 30;11:e2983. doi: 10.7717/peerj-cs.2983 (PMC12453852; doi:10.7717/peerj-cs.2983)
Supplement: Supplemental Information 6 — Instructions on setting up the development environment, organizing code files, and executing experiments to ensure reproducibility. [file peerj-cs-11-2983-s006.docx]

**Deep Context-Attentive Transformer Transfer Learning for Financial Forecasting**

Ling Feng^1^, Ananta Sinchai^1^

^1^ School of Integrated Innovative Technology, King Mongkut’s Institute of Technology, Ladkrabang, Bangkok, Thailand

Corresponding Author:

Ananta Sinchai^2^

Chalong Krung Road, Ladkrabang, Bangkok, 10520, Thailand

Email address: ananta.sin@kmitl.ac.th

Contributing Author:

Ling Feng^1^

Chalong Krung Road, Ladkrabang, Bangkok, 10520, Thailand

Email address: 64612007@kmitl.ac.th

**SUPPLEMENTARY INFORMATION**

This supplementary information document provides a comprehensive guide for reproducing the proposed Deep Context-Attentive Transformer Transfer Learning model for financial forecasting. Reproducing includes detailed instructions on setting up the environment, required libraries, dataset structure, and code organization.

Key sections:

- **Reproduction Procedures:** Instructions on setting up Python-based environments and dependencies for running the model.
- **Organizational Structure:** Explanation of folder and file hierarchies within the project, ensuring reproducibility.
- **Code Overview:** Description of the core components, including data handling scripts (data_loader.py, data_factory.py), experimental setup (exp_basic.py, exp_main.py), and utilities (masking.py, metrics.py, tools.py).
- **Dataset Structure:** Guidelines for formatting data input, particularly financial time-series, including Open, High, Low, and Closing (OT) prices.
- **Model Implementation:** Details of the CNNCorrelationBasedTransformer model, highlighting its CNN correlation-based attention mechanism for time-series forecasting regarding context-attentive Transformer.

This document serves as a technical reference for researchers and developers aiming to replicate or extend the study.

**1. REPRODUCTION PROCEDURES**

In this section, we describe how to reproduce the proposed method using the modified code based on a Python interpreter. The original code developed by Haixu Wu, available on [GitHub](https://github.com/thuml/Autoformer), has been adapted to serve the requirements of our experiments.

All modifications to the original code were made in accordance with the MIT License below:

MIT License

Copyright (c) 2021 THUML @ Tsinghua University

Permission is hereby granted, free of charge, to any person obtaining a copy of this software and associated documentation files (the "Software"), to deal in the Software without restriction, including without limitation the rights to use, copy, modify, merge, publish, distribute, sublicense, and/or sell copies of the Software, and to permit persons to whom the Software is furnished to do so, subject to the following conditions:

The above copyright notice and this permission notice shall be included in all copies or substantial portions of the Software.

THE SOFTWARE IS PROVIDED "AS IS", WITHOUT WARRANTY OF ANY KIND, EXPRESS OR IMPLIED, INCLUDING BUT NOT LIMITED TO THE WARRANTIES OF MERCHANTABILITY, FITNESS FOR A PARTICULAR PURPOSE AND NONINFRINGEMENT. IN NO EVENT SHALL THE AUTHORS OR COPYRIGHT HOLDERS BE LIABLE FOR ANY CLAIM, DAMAGES OR OTHER LIABILITY, WHETHER IN AN ACTION OF CONTRACT, TORT OR OTHERWISE, ARISING FROM, OUT OF OR IN CONNECTION WITH THE SOFTWARE OR THE USE OR OTHER DEALINGS IN THE SOFTWARE.

1.1 Prerequisite requirements

This subsection outlines the prerequisites for setting up a development environment for machine learning projects using PyTorch. It details the required Python version and necessary libraries, ensuring you can successfully run your code and experiment with the proposed model. Follow the steps to configure the environment effectively.

1.1.1 Python: Ensure Python 3.6 or higher is installed.

1.1.2 Jupyter: Ensure JupyterLab 4.2 or higher is installed.

1.1.3 Install Required Libraries:

pip install torch torchvision torchaudio

pip install numpy pandas matplotlib scikit-learn argparse

1.1.4 Custom Utilities: Ensure that any custom utility modules (utils/timefeatures.py, utils/tools.py, etc.) are available in your project directory.

1.2 Organizational structure of folders and files

To reproduce the experiment, a specific set of folders and files organized in a hierarchical data structure is required (see Figure S1). Developers—including users, scholars, or researchers are suggested to make sure that these folders and files are produced properly. Upon running Execute.ipynb, additional folders and files will be automatically created in the MyProject root directory. These automatically generated items will include at least “checkpoints,” “test_results,” and “result.txt.” The “checkpoints” folder is specifically designed to collect a series of parameters, storing the obtained parameter settings in files with a “.pth” extension for each experiment. This setup supports transfer learning by allowing pre-trained models to be fine-tuned on new tasks, enhancing their performance and adaptability. Additionally, other folders and files may be generated by Jupyter that are not explicitly mentioned here.

/MyProject

├── /data_provider

│ ├── __init__.py

│ ├── data_factory.py

│ └── data_loader.py

├── /data

│ ├── dataset_instrument01.csv

│ ├── …

│ ├── …

│ ├── …

│ └── dataset_instrumentNN.csv

├── /exp

│ ├── __init__.py

│ ├── exp_basic.py

│ └── exp_main.py

├── /utils

│ ├── __init__.py

│ ├── masking.py

│ ├── metrics.py

│ ├── timefeatures.py

│ └── tools.py

├── /results

├── CNNCorrelationBasedTransformer.py

└── Execute.ipynb

**Figure S1** Hierarchical structure of the MyProject directory.

This diagram depicts the hierarchical organization of the MyProject directory. It includes essential subfolders like /data_provider, /data, /exp, and /utils, each containing necessary scripts and datasets. Following the execution of Execute.ipynb, additional folders such as “checkpoints,” “test_results,” and “result.txt” are automatically generated in the root directory, along with other files potentially created by Jupyter. This structure facilitates efficient data management and reproducibility of the experiment.

1.3 Overview of code structure, functionality, and file preparation

In this section, we provide a detailed overview of the code contained within each file of the MyProject directory. This organized structure, illustrated in Figure S1, facilitates efficient data management and experimentation. Each file and folder are designed to serve a specific purpose within this work, from data handling to experimental execution.

The /data_provider folder contains scripts essential for loading and preparing datasets, while the /data folder houses the various datasets utilized in our experiments. The /exp directory is dedicated to the core experimental code, including the main execution script, Execute.ipynb. The /utils folder comprises utility functions that enhance the overall functionality of the proposed method.

A key component of this work is the CNNCorrelationBasedTransformer.py file, which includes significant classes that play a crucial role in the experimental framework. By breaking down the code for each file, we aim to clarify the role and functionality of every component, ensuring that users can easily navigate and reproduce the methods outlined in the following parts of this study.

1.3.1 Dataset structure in a CSV file format

To create a dataset consisting of five attributes to be consistent to the use in this work, which includes financial metrics, follow these steps to collect and structure your data:

**Date**: Ensure that dates are in a consistent format (e.g., YYYY-MM-DD). These dates typically represent trading days, as weekends and holidays might not have data. Such data can be obtained from historical financial data providers, stock market APIs, or other financial data sources.

**Open, High, Low, Closing (OT)**: These values represent the daily trading range:

**Open**: The opening price at the start of the trading day

**High**: The highest price recorded during the trading day

**Low**: The lowest price recorded during the trading day

**Closing (OT)**: The price at which the instrument closed at the end of the trading day

Each row in the CSV file should represent one trading day, maintaining consistency in format and completeness across all entries. Collect data over a range that matches your intended analysis, whether it is daily, weekly, or at other intervals. Please keep in mind that this is not limited to financial time series data; other types of time series data can also be used. Additionally, ensure that the last column (OT) represents the objective target feature to predict.

1.3.1.1 Example CSV Structure

An example CSV structure is shown in Figure S2. This illustration captures how each of the metrics is arranged within the CSV. Ensure that the created file follows this example for uniformity and compatibility with existing datasets or analysis tools.

To maintain data quality, verify each entry against reliable sources. For financial datasets, cross-referencing with multiple data providers can help maintain accuracy, especially for historical values. Proper formatting, as shown in Figure S2, also ensures that your dataset remains compatible with statistical software and machine learning algorithms.


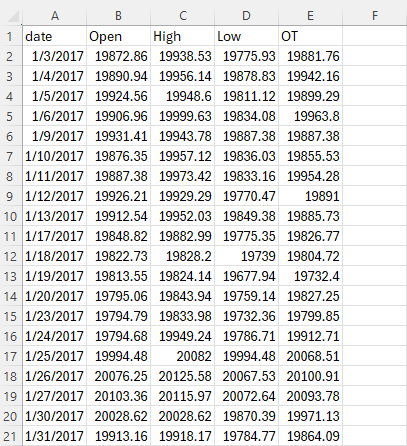


**Figure S2** Example of a CSV dataset structure for time series forecasting in finance, showing columns for date, Open, High, Low, and closing price (OT). Each row represents a trading day, capturing essential daily price metrics, with OT as the closing value. This format supports consistent data input for forecasting and financial modeling.

1.3.2 Generation of __init__.py

The __init__.py file is a crucial component in Python package creation, indicating to the interpreter that a directory should be treated as a package. When a new package is generated, many development environments and frameworks automatically create a blank __init__.py file. This blank file serves as a placeholder, simplifying the package structure without requiring any additional code. Developers—including users, scholars, or researchers—must include this file if creating a package manually to ensure proper functionality and avoid import issues, even though it does not contain any code by default.

1.3.3 Generation of data_factory.py

This code (see below) implements a data loading system primarily designed for time series datasets. The core functionality revolves around the data_provider function, which creates data loaders for different types of financial market data (stocks, cryptocurrencies, commodities, etc.).

The code uses a dictionary data_dict to map various dataset identifiers (like 'btc' for Bitcoin, 'sp' for S&P 500, etc.) to a custom dataset class called Dataset_Custom. The data_provider function accepts arguments for configuration and a flag parameter ('test', 'pred', or other) that determines how the data will be loaded. For testing, it disables shuffling and keeps all batches. For prediction, it sets batch size to 1. For training (default), it enables shuffling and drops incomplete final batches. Developers—including users, scholars, or researchers may copy the given code and paste it in a file named data_factory.py and save it.

#-----------------------------------------------------------data_factory.py--------------------------------------#

from data_provider.data_loader import Dataset_Custom, Dataset_Pred

from torch.utils.data import DataLoader

data_dict = {

'custom': Dataset_Custom,

'dj': Dataset_Custom,

'sp': Dataset_Custom,

'nq': Dataset_Custom,

'nk': Dataset_Custom,

'dax': Dataset_Custom,

'sp1': Dataset_Custom,

'nk1': Dataset_Custom,

'dax1': Dataset_Custom,

'gold': Dataset_Custom,

'gold1': Dataset_Custom,

'btc': Dataset_Custom,

'btc1': Dataset_Custom,

'eth': Dataset_Custom,

'eth1': Dataset_Custom,

'eth2': Dataset_Custom,

'set': Dataset_Custom,

'set1': Dataset_Custom,

'dj2': Dataset_Custom,

'oil': Dataset_Custom,

'oil1': Dataset_Custom,

'ibo': Dataset_Custom,

'ibo1': Dataset_Custom,

'sensex': Dataset_Custom,

'sensex1': Dataset_Custom,

'ltc': Dataset_Custom,

'ltc1': Dataset_Custom,

'nq1': Dataset_Custom,

}

def data_provider(args, flag):

Data = data_dict[args.data]

timeenc = 0 if args.embed != 'timeF' else 1

if flag == 'test':

shuffle_flag = False

drop_last = False

batch_size = args.batch_size

freq = args.freq

elif flag == 'pred':

shuffle_flag = False

drop_last = False

batch_size = 1

freq = args.freq

Data = Dataset_Pred

else:

shuffle_flag = True

drop_last = True

batch_size = args.batch_size

freq = args.freq

data_set = Data(

root_path=args.root_path,

data_path=args.data_path,

flag=flag,

size=[args.seq_len, args.label_len, args.pred_len],

features=args.features,

target=args.target,

timeenc=timeenc,

freq=freq

)

print(flag, len(data_set))

data_loader = DataLoader(

data_set,

batch_size=batch_size,

shuffle=shuffle_flag,

num_workers=args.num_workers,

drop_last=drop_last)

return data_set, data_loader

#----------------------------------------------------------data_factory.py---------------------------------------#

1.3.4 Generation of data_loader.py

This implementation provides a PyTorch-based data loading system specifically designed for time series forecasting. The system consists of two main components:

1. Dataset_Custom: The primary dataset class for training/testing, which handles:
   - Data splitting (70% train, 20% test, 10% validation)
   - Time feature encoding (date features or custom time encodings)
   - Data normalization using StandardScaler
   - Flexible sequence length configuration for input, label, and prediction windows
   - Support for both single-feature (S) and multi-feature (M/MS) time series
2. Dataset_Pred: A specialized version for prediction that:
   - Uses the last available sequence for forecasting.
   - Generates future timestamp features for prediction period.
   - Supports inverse transformation of scaled data.

The data factory (data_factory.py) acts as a wrapper that:

- Maps dataset names to appropriate dataset classes.
- Configures DataLoader with correct parameters for training, testing, or prediction.
- Handles batch size, shuffling, and worker settings based on the usage mode.

To use this system:

1. Prepare your time series data in CSV format with a 'date' column and target variable.
2. Configure parameters (sequence length, prediction length, features type).
3. Use data_provider() to get appropriate data loaders for training/testing/prediction.

The system is particularly optimized for financial time series like stocks, cryptocurrencies, and commodities, as evidenced by the dataset mappings in data_factory.py. Developers—including users, scholars, or researchers may copy the given code and paste it in a file named data_loader.py and save it.

#----------------------------------------------------------data_loader.py----------------------------------------#

import os

import numpy as np

import pandas as pd

import os

import torch

from torch.utils.data import Dataset, DataLoader

from sklearn.preprocessing import StandardScaler

from utils.timefeatures import time_features

import warnings

warnings.filterwarnings('ignore')

class Dataset_Custom(Dataset):

def __init__(self, root_path, flag='train', size=None,

features='MS', data_path='ibo.csv',

target='OT', scale=True, timeenc=0, freq='d'):

# size [seq_len, label_len, pred_len]

# info

if size == None:

self.seq_len = 24 * 4 * 4

self.label_len = 24 * 4

self.pred_len = 24 * 4

else:

self.seq_len = size[0]

self.label_len = size[1]

self.pred_len = size[2]

# init

assert flag in ['train', 'test', 'val']

type_map = {'train': 0, 'val': 1, 'test': 2}

self.set_type = type_map[flag]

self.features = features

self.target = target

self.scale = scale

self.timeenc = timeenc

self.freq = freq

self.root_path = root_path

self.data_path = data_path

self.__read_data__()

def __read_data__(self):

self.scaler = StandardScaler()

df_raw = pd.read_csv(os.path.join(self.root_path,

self.data_path))

'''

df_raw.columns: ['date', ...(other features), target feature]

'''

cols = list(df_raw.columns)

cols.remove(self.target)

cols.remove('date')

df_raw = df_raw[['date'] + cols + [self.target]]

# print(cols)

num_train = int(len(df_raw) * 0.7)

num_test = int(len(df_raw) * 0.2)

num_vali = len(df_raw) - num_train - num_test

border1s = [0, num_train - self.seq_len, len(df_raw) - num_test - self.seq_len]

border2s = [num_train, num_train + num_vali, len(df_raw)]

border1 = border1s[self.set_type]

border2 = border2s[self.set_type]

if self.features == 'M' or self.features == 'MS':

cols_data = df_raw.columns[1:]

df_data = df_raw[cols_data]

elif self.features == 'S':

df_data = df_raw[[self.target]]

if self.scale:

train_data = df_data[border1s[0]:border2s[0]]

self.scaler.fit(train_data.values)

data = self.scaler.transform(df_data.values)

else:

data = df_data.values

df_stamp = df_raw[['date']][border1:border2]

df_stamp['date'] = pd.to_datetime(df_stamp.date)

if self.timeenc == 0:

df_stamp['month'] = df_stamp.date.apply(lambda row: row.month, 1)

df_stamp['day'] = df_stamp.date.apply(lambda row: row.day, 1)

df_stamp['weekday'] = df_stamp.date.apply(lambda row: row.weekday(), 1)

df_stamp['hour'] = df_stamp.date.apply(lambda row: row.hour, 1)

data_stamp = df_stamp.drop(['date'], 1).values

elif self.timeenc == 1:

data_stamp = time_features(pd.to_datetime(df_stamp['date'].values), freq=self.freq)

data_stamp = data_stamp.transpose(1, 0)

self.data_x = data[border1:border2]

self.data_y = data[border1:border2]

self.data_stamp = data_stamp

def __getitem__(self, index):

s_begin = index

s_end = s_begin + self.seq_len

r_begin = s_end - self.label_len

r_end = r_begin + self.label_len + self.pred_len

seq_x = self.data_x[s_begin:s_end]

seq_y = self.data_y[r_begin:r_end]

seq_x_mark = self.data_stamp[s_begin:s_end]

seq_y_mark = self.data_stamp[r_begin:r_end]

return seq_x, seq_y, seq_x_mark, seq_y_mark

def __len__(self):

return len(self.data_x) - self.seq_len - self.pred_len + 1

def inverse_transform(self, data):

return self.scaler.inverse_transform(data)

class Dataset_Pred(Dataset):

def __init__(self, root_path, flag='pred', size=None,

features='S', data_path='ETTh1.csv',

target='OT', scale=True, inverse=False, timeenc=0, freq='15min', cols=None):

# size [seq_len, label_len, pred_len]

# info

if size == None:

self.seq_len = 24 * 4 * 4

self.label_len = 24 * 4

self.pred_len = 24 * 4

else:

self.seq_len = size[0]

self.label_len = size[1]

self.pred_len = size[2]

# init

assert flag in ['pred']

self.features = features

self.target = target

self.scale = scale

self.inverse = inverse

self.timeenc = timeenc

self.freq = freq

self.cols = cols

self.root_path = root_path

self.data_path = data_path

self.__read_data__()

def __read_data__(self):

self.scaler = StandardScaler()

df_raw = pd.read_csv(os.path.join(self.root_path,

self.data_path))

'''

df_raw.columns: ['date', ...(other features), target feature]

'''

if self.cols:

cols = self.cols.copy()

cols.remove(self.target)

else:

cols = list(df_raw.columns)

cols.remove(self.target)

cols.remove('date')

df_raw = df_raw[['date'] + cols + [self.target]]

border1 = len(df_raw) - self.seq_len

border2 = len(df_raw)

if self.features == 'M' or self.features == 'MS':

cols_data = df_raw.columns[1:]

df_data = df_raw[cols_data]

elif self.features == 'S':

df_data = df_raw[[self.target]]

if self.scale:

self.scaler.fit(df_data.values)

data = self.scaler.transform(df_data.values)

else:

data = df_data.values

tmp_stamp = df_raw[['date']][border1:border2]

tmp_stamp['date'] = pd.to_datetime(tmp_stamp.date)

pred_dates = pd.date_range(tmp_stamp.date.values[-1], periods=self.pred_len + 1, freq=self.freq)

df_stamp = pd.DataFrame(columns=['date'])

df_stamp.date = list(tmp_stamp.date.values) + list(pred_dates[1:])

if self.timeenc == 0:

df_stamp['month'] = df_stamp.date.apply(lambda row: row.month, 1)

df_stamp['day'] = df_stamp.date.apply(lambda row: row.day, 1)

df_stamp['weekday'] = df_stamp.date.apply(lambda row: row.weekday(), 1)

df_stamp['hour'] = df_stamp.date.apply(lambda row: row.hour, 1)

df_stamp['minute'] = df_stamp.date.apply(lambda row: row.minute, 1)

df_stamp['minute'] = df_stamp.minute.map(lambda x: x // 15)

data_stamp = df_stamp.drop(['date'], 1).values

elif self.timeenc == 1:

data_stamp = time_features(pd.to_datetime(df_stamp['date'].values), freq=self.freq)

data_stamp = data_stamp.transpose(1, 0)

self.data_x = data[border1:border2]

if self.inverse:

self.data_y = df_data.values[border1:border2]

else:

self.data_y = data[border1:border2]

self.data_stamp = data_stamp

def __getitem__(self, index):

s_begin = index

s_end = s_begin + self.seq_len

r_begin = s_end - self.label_len

r_end = r_begin + self.label_len + self.pred_len

seq_x = self.data_x[s_begin:s_end]

if self.inverse:

seq_y = self.data_x[r_begin:r_begin + self.label_len]

else:

seq_y = self.data_y[r_begin:r_begin + self.label_len]

seq_x_mark = self.data_stamp[s_begin:s_end]

seq_y_mark = self.data_stamp[r_begin:r_end]

return seq_x, seq_y, seq_x_mark, seq_y_mark

def __len__(self):

return len(self.data_x) - self.seq_len + 1

def inverse_transform(self, data):

return self.scaler.inverse_transform(data)

#----------------------------------------------------------data_loader.py----------------------------------------#

1.3.5 Generation of exp_basic.py

The code implements a basic experimental framework for time series forecasting, with an emphasis on financial data. At its core, Exp_Basic serves as an abstract base class that provides fundamental experiment management functionality:

- Device management (CPU/GPU selection)
- Model initialization
- Template methods for training, testing, and validation

Developers—including users, scholars, or researchers may copy the given code and paste it in a file named exp_basic.py and save it. This base class is designed to be extended by specific experiment implementations.

The data loading system consists of:

1. A data factory (data_factory.py) that manages dataset creation and loading configurations.
2. Custom dataset classes (data_loader.py) that handle:
   - Data preprocessing and normalization
   - Time feature engineering
   - Sequence preparation for both training and prediction
   - Train/validation/test splits (70/10/20)

This system is particularly suited for financial time series analysis, supporting various markets (stocks, crypto, commodities) with flexible input features and prediction windows. The modular design allows for easy extension to new datasets while maintaining consistent data handling across experiments.

To use this framework, users should:

1. Set up the appropriate directory structure.
2. Place their time series data in CSV format.
3. Extend the Exp_Basic class for their specific experiment needs.
4. Configure data loading parameters through the args system.

The framework handles the complexities of data preparation and device management, allowing researchers to focus on model implementation and experimentation.

#----------------------------------------------------------exp_basic.py------------------------------------------#

import os

import torch

import numpy as np

class Exp_Basic(object):

def __init__(self, args):

self.args = args

self.device = self._acquire_device()

self.model = self._build_model().to(self.device)

def _build_model(self):

raise NotImplementedError

return None

def _acquire_device(self):

if self.args.use_gpu:

os.environ["CUDA_VISIBLE_DEVICES"] = str(

self.args.gpu) if not self.args.use_multi_gpu else self.args.devices

device = torch.device('cuda:{}'.format(self.args.gpu))

print('Use GPU: cuda:{}'.format(self.args.gpu))

else:

device = torch.device('cpu')

print('Use CPU')

return device

def _get_data(self):

pass

def vali(self):

pass

def train(self):

pass

def test(self):

pass

#----------------------------------------------------------exp_basic.py------------------------------------------#

1.3.6 Generation of exp_main.py

The code implements a complete experimental framework for time series forecasting, particularly focused on financial data. The system is structured with clear separation of concerns:

1. exp_basic.py - The abstract base class providing fundamental experiment infrastructure:
   - Device management (CPU/GPU)
   - Model initialization
   - Template methods for core functionality
2. exp_main.py - The main experiment implementation that extends Exp_Basic:
   - Implements training, validation, and testing loops.
   - Handles model optimization and loss calculation.
   - Supports mixed-precision training with AMP.
   - Includes early stopping and learning rate adjustment.
   - Provides comprehensive metrics calculation and result saving.
   - Implements prediction functionality for real-world forecasting.
3. Data Management System:
   - data_factory.py: Manages dataset creation and configuration.
   - data_loader.py: Handles data preprocessing, normalization, and sequence preparation.

The system supports:

- Multiple financial markets (stocks, crypto, commodities)
- Flexible sequence lengths for input/prediction
- Both single and multi-feature time series
- Automated train/validation/test splits
- Comprehensive result logging and visualization
- GPU acceleration with multi-GPU support
- Mixed precision training for improved performance

This implementation particularly focuses on transformer-based models with correlation mechanisms, providing a robust framework for financial time series forecasting research and applications. Developers—including users, scholars, or researchers may copy the given code and paste it in a file named exp_main.py and save it.

#----------------------------------------------------------exp_main.py------------------------------------------#

from data_provider.data_factory import data_provider

from exp.exp_basic import Exp_Basic

import CNNCorrelationBasedTransformer

from utils.tools import EarlyStopping, adjust_learning_rate, visual

from utils.metrics import metric

import numpy as np

import torch

import torch.nn as nn

from torch import optim

import os

import time

import warnings

import matplotlib.pyplot as plt

import numpy as np

warnings.filterwarnings('ignore')

class Exp_Main(Exp_Basic):

def __init__(self, args):

super(Exp_Main, self).__init__(args)

def _build_model(self):

model_dict = {

'CNNCorrelationBasedTransformer': CNNCorrelationBasedTransformer,

}

model = model_dict[self.args.model].Model(self.args).float()

if self.args.use_multi_gpu and self.args.use_gpu:

model = nn.DataParallel(model, device_ids=self.args.device_ids)

return model

def _get_data(self, flag):

data_set, data_loader = data_provider(self.args, flag)

return data_set, data_loader

def _select_optimizer(self):

model_optim = optim.Adam(self.model.parameters(), lr=self.args.learning_rate)

return model_optim

def _select_criterion(self):

criterion = nn.MSELoss()

return criterion

def vali(self, vali_data, vali_loader, criterion):

total_loss = []

self.model.eval()

with torch.no_grad():

for i, (batch_x, batch_y, batch_x_mark, batch_y_mark) in enumerate(vali_loader):

batch_x = batch_x.float().to(self.device)

batch_y = batch_y.float()

batch_x_mark = batch_x_mark.float().to(self.device)

batch_y_mark = batch_y_mark.float().to(self.device)

# decoder input

dec_inp = torch.zeros_like(batch_y[:, -self.args.pred_len:, :]).float()

dec_inp = torch.cat([batch_y[:, :self.args.label_len, :], dec_inp], dim=1).float().to(self.device)

# encoder - decoder

if self.args.use_amp:

with torch.cuda.amp.autocast():

if self.args.output_attention:

outputs = self.model(batch_x, batch_x_mark, dec_inp, batch_y_mark)[0]

else:

outputs = self.model(batch_x, batch_x_mark, dec_inp, batch_y_mark)

else:

if self.args.output_attention:

outputs = self.model(batch_x, batch_x_mark, dec_inp, batch_y_mark)[0]

else:

outputs = self.model(batch_x, batch_x_mark, dec_inp, batch_y_mark)

f_dim = -1 if self.args.features == 'MS' else 0

outputs = outputs[:, -self.args.pred_len:, f_dim:]

batch_y = batch_y[:, -self.args.pred_len:, f_dim:].to(self.device)

pred = outputs.detach().cpu()

true = batch_y.detach().cpu()

loss = criterion(pred, true)

total_loss.append(loss)

total_loss = np.average(total_loss)

self.model.train()

return total_loss

def train(self, setting):

train_data, train_loader = self._get_data(flag='train')

vali_data, vali_loader = self._get_data(flag='val')

test_data, test_loader = self._get_data(flag='test')

path = os.path.join(self.args.checkpoints, setting)

if not os.path.exists(path):

os.makedirs(path)

time_now = time.time()

train_steps = len(train_loader)

early_stopping = EarlyStopping(patience=self.args.patience, verbose=True)

model_optim = self._select_optimizer()

criterion = self._select_criterion()

if self.args.use_amp:

scaler = torch.cuda.amp.GradScaler()

for epoch in range(self.args.train_epochs):

iter_count = 0

train_loss = []

self.model.train()

epoch_time = time.time()

for i, (batch_x, batch_y, batch_x_mark, batch_y_mark) in enumerate(train_loader):

iter_count += 1

model_optim.zero_grad()

batch_x = batch_x.float().to(self.device)

batch_y = batch_y.float().to(self.device)

batch_x_mark = batch_x_mark.float().to(self.device)

batch_y_mark = batch_y_mark.float().to(self.device)

# decoder input

dec_inp = torch.zeros_like(batch_y[:, -self.args.pred_len:, :]).float()

dec_inp = torch.cat([batch_y[:, :self.args.label_len, :], dec_inp], dim=1).float().to(self.device)

# encoder - decoder

if self.args.use_amp:

with torch.cuda.amp.autocast():

if self.args.output_attention:

outputs = self.model(batch_x, batch_x_mark, dec_inp, batch_y_mark)[0]

else:

outputs = self.model(batch_x, batch_x_mark, dec_inp, batch_y_mark)

f_dim = -1 if self.args.features == 'MS' else 0

outputs = outputs[:, -self.args.pred_len:, f_dim:]

batch_y = batch_y[:, -self.args.pred_len:, f_dim:].to(self.device)

loss = criterion(outputs, batch_y)

train_loss.append(loss.item())

else:

if self.args.output_attention:

outputs = self.model(batch_x, batch_x_mark, dec_inp, batch_y_mark)[0]

else:

outputs = self.model(batch_x, batch_x_mark, dec_inp, batch_y_mark)

f_dim = -1 if self.args.features == 'MS' else 0

outputs = outputs[:, -self.args.pred_len:, f_dim:]

batch_y = batch_y[:, -self.args.pred_len:, f_dim:].to(self.device)

loss = criterion(outputs, batch_y)

train_loss.append(loss.item())

if (i + 1) % 100 == 0:

print("\titers: {0}, epoch: {1} | loss: {2:.7f}".format(i + 1, epoch + 1, loss.item()))

speed = (time.time() - time_now) / iter_count

left_time = speed * ((self.args.train_epochs - epoch) * train_steps - i)

print('\tspeed: {:.4f}s/iter; left time: {:.4f}s'.format(speed, left_time))

iter_count = 0

time_now = time.time()

if self.args.use_amp:

scaler.scale(loss).backward()

scaler.step(model_optim)

scaler.update()

else:

loss.backward()

model_optim.step()

print("Epoch: {} cost time: {}".format(epoch + 1, time.time() - epoch_time))

train_loss = np.average(train_loss)

vali_loss = self.vali(vali_data, vali_loader, criterion)

test_loss = self.vali(test_data, test_loader, criterion)

print("Epoch: {0}, Steps: {1} | Train Loss: {2:.7f} Vali Loss: {3:.7f} Test Loss: {4:.7f}".format(

epoch + 1, train_steps, train_loss, vali_loss, test_loss))

early_stopping(vali_loss, self.model, path)

if early_stopping.early_stop:

print("Early stopping")

break

adjust_learning_rate(model_optim, epoch + 1, self.args)

best_model_path = path + '/' + 'checkpoint.pth'

self.model.load_state_dict(torch.load(best_model_path))

return

def test(self, setting, test=0):

test_data, test_loader = self._get_data(flag='test')

if test:

print('loading model')

self.model.load_state_dict(torch.load(os.path.join('./checkpoints/' + setting, 'checkpoint.pth')))

preds = []

trues = []

folder_path = './test_results/' + setting + '/'

if not os.path.exists(folder_path):

os.makedirs(folder_path)

self.model.eval()

with torch.no_grad():

for i, (batch_x, batch_y, batch_x_mark, batch_y_mark) in enumerate(test_loader):

batch_x = batch_x.float().to(self.device)

batch_y = batch_y.float().to(self.device)

batch_x_mark = batch_x_mark.float().to(self.device)

batch_y_mark = batch_y_mark.float().to(self.device)

# decoder input

dec_inp = torch.zeros_like(batch_y[:, -self.args.pred_len:, :]).float()

dec_inp = torch.cat([batch_y[:, :self.args.label_len, :], dec_inp], dim=1).float().to(self.device)

# encoder - decoder

if self.args.use_amp:

with torch.cuda.amp.autocast():

if self.args.output_attention:

outputs = self.model(batch_x, batch_x_mark, dec_inp, batch_y_mark)[0]

else:

outputs = self.model(batch_x, batch_x_mark, dec_inp, batch_y_mark)

else:

if self.args.output_attention:

outputs = self.model(batch_x, batch_x_mark, dec_inp, batch_y_mark)[0]

else:

outputs = self.model(batch_x, batch_x_mark, dec_inp, batch_y_mark)

f_dim = -1 if self.args.features == 'MS' else 0

outputs = outputs[:, -self.args.pred_len:, f_dim:]

batch_y = batch_y[:, -self.args.pred_len:, f_dim:].to(self.device)

outputs = outputs.detach().cpu().numpy()

batch_y = batch_y.detach().cpu().numpy()

pred = outputs # outputs.detach().cpu().numpy() # .squeeze()

true = batch_y # batch_y.detach().cpu().numpy() # .squeeze()

preds.append(pred)

trues.append(true)

if i % 20 == 0:

input = batch_x.detach().cpu().numpy()

gt = np.concatenate((input[0, :, -1], true[0, :, -1]), axis=0)

pd = np.concatenate((input[0, :, -1], pred[0, :, -1]), axis=0)

visual(gt, pd, os.path.join(folder_path, str(i) + '.pdf'))

preds = np.concatenate(preds, axis=0)

trues = np.concatenate(trues, axis=0)

print('test shape:', preds.shape, trues.shape)

preds = preds.reshape(-1, preds.shape[-2], preds.shape[-1])

trues = trues.reshape(-1, trues.shape[-2], trues.shape[-1])

print('test shape:', preds.shape, trues.shape)

# result save

folder_path = './results/' + setting + '/'

if not os.path.exists(folder_path):

os.makedirs(folder_path)

mae, mse, rmse, mape, mspe, R2, rse = metric(preds, trues)

print('mse:{}, mae:{}'.format(mse, mae))

f = open("result.txt", 'a')

f.write(setting + " \n")

f.write('mse:{}, mae:{}'.format(mse, mae))

f.write('\n')

f.write('\n')

f.close()

np.save(folder_path + 'metrics.npy', np.array([mae, mse, rmse, mape, mspe, R2, rse]))

np.save(folder_path + 'pred.npy', preds)

np.save(folder_path + 'true.npy', trues)

return

def predict(self, setting, load=False):

pred_data, pred_loader = self._get_data(flag='pred')

if load:

path = os.path.join(self.args.checkpoints, setting)

best_model_path = path + '/' + 'checkpoint.pth'

self.model.load_state_dict(torch.load(best_model_path))

preds = []

self.model.eval()

with torch.no_grad():

for i, (batch_x, batch_y, batch_x_mark, batch_y_mark) in enumerate(pred_loader):

batch_x = batch_x.float().to(self.device)

batch_y = batch_y.float()

batch_x_mark = batch_x_mark.float().to(self.device)

batch_y_mark = batch_y_mark.float().to(self.device)

# decoder input

dec_inp = torch.zeros([batch_y.shape[0], self.args.pred_len, batch_y.shape[2]]).float()

dec_inp = torch.cat([batch_y[:, :self.args.label_len, :], dec_inp], dim=1).float().to(self.device)

# encoder - decoder

if self.args.use_amp:

with torch.cuda.amp.autocast():

if self.args.output_attention:

outputs = self.model(batch_x, batch_x_mark, dec_inp, batch_y_mark)[0]

else:

outputs = self.model(batch_x, batch_x_mark, dec_inp, batch_y_mark)

else:

if self.args.output_attention:

outputs = self.model(batch_x, batch_x_mark, dec_inp, batch_y_mark)[0]

else:

outputs = self.model(batch_x, batch_x_mark, dec_inp, batch_y_mark)

pred = outputs.detach().cpu().numpy() # .squeeze()

preds.append(pred)

preds = np.array(preds)

preds = preds.reshape(-1, preds.shape[-2], preds.shape[-1])

# result save

folder_path = './results/' + setting + '/'

if not os.path.exists(folder_path):

os.makedirs(folder_path)

np.save(folder_path + 'real_prediction.npy', preds)

return

#----------------------------------------------------------exp_main.py------------------------------------------#

1.3.7 Generation of masking.py

This code implements two essential masking classes for attention mechanisms in transformer-based models, particularly for time series forecasting:

1. TriangularCausalMask: Creates a causal (triangular) attention mask that ensures each position can only attend to previous positions and itself. This is crucial for maintaining the temporal causality in time series predictions - preventing information leakage from future to past timesteps.
2. ProbMask: Implements a probabilistic mask for attention scores, typically used in probabilistic attention mechanisms. It combines triangular masking with specific attention patterns based on provided indices and scores, allowing for more sophisticated attention patterns while maintaining causality.

Both classes use PyTorch's efficient tensor operations and support both CPU and GPU execution through the device parameter. The masks are implemented as boolean tensors where True values indicate positions that should be masked (ignored) in the attention computation.

Developers can copy this code into a file named masking.py and import these classes for implementing attention mechanisms in their transformer-based models. The classes are particularly designed for sequence modeling tasks where maintaining temporal causality is important.

#-----------------------------------------------------------masking.py-------------------------------------------#

import torch

class TriangularCausalMask():

def __init__(self, B, L, device="cpu"):

mask_shape = [B, 1, L, L]

with torch.no_grad():

self._mask = torch.triu(torch.ones(mask_shape, dtype=torch.bool), diagonal=1).to(device)

@property

def mask(self):

return self._mask

class ProbMask():

def __init__(self, B, H, L, index, scores, device="cpu"):

_mask = torch.ones(L, scores.shape[-1], dtype=torch.bool).to(device).triu(1)

_mask_ex = _mask[None, None, :].expand(B, H, L, scores.shape[-1])

indicator = _mask_ex[torch.arange(B)[:, None, None],

torch.arange(H)[None, :, None],

index, :].to(device)

self._mask = indicator.view(scores.shape).to(device)

@property

def mask(self):

return self._mask

#-----------------------------------------------------------masking.py-------------------------------------------#

1.3.8 Generation of metrics.py

This code provides a comprehensive collection of common evaluation metrics for comparing predicted values against true (actual) values, primarily used in regression and forecasting tasks. The file implements eight key metrics: Mean Absolute Error (MAE), Mean Squared Error (MSE), Root Mean Squared Error (RMSE), Mean Absolute Percentage Error (MAPE), Mean Squared Percentage Error (MSPE), R-squared (R²), Mean Hassanat Distance (MHD), and Relative Squared Error (RSE). Additionally, it includes a correlation coefficient (CORR) calculation.

The main function metric() serves as a convenience wrapper that returns all primary metrics in a single call. To use this code, users need to have NumPy installed and should pass their predicted and true values as NumPy arrays of matching shapes. All metrics are implemented to handle both single-dimensional and multi-dimensional arrays, making them suitable for various prediction tasks.

To reproduce this functionality, users can simply copy the provided code into a file named 'metrics.py' and import it into their projects. The only dependency required is NumPy. All functions expect two arguments: pred (predicted values) and true (actual values), both as NumPy arrays.

#------------------------------------------------------------metrics.py-------------------------------------------#

import numpy as np

def RSE(pred, true):

return np.sqrt(np.sum((true - pred) ** 2)) / np.sqrt(np.sum((true - true.mean()) ** 2))

def CORR(pred, true):

u = ((true - true.mean(0)) * (pred - pred.mean(0))).sum(0)

d = np.sqrt(((true - true.mean(0)) ** 2).sum(0) * ((pred - pred.mean(0)) ** 2).sum(0))

return (u / d).mean(-1)

def MAE(pred, true):

return np.mean(np.abs(pred - true))

def MSE(pred, true):

return np.mean((pred - true) ** 2)

def RMSE(pred, true):

return np.sqrt(MSE(pred, true))

def MAPE(pred, true):

return np.mean(np.abs((pred - true) / true))

def MSPE(pred, true):

return np.mean(np.square((pred - true) / true))

def MHD(preds, trues):

pred = preds.reshape(-1)

true = trues.reshape(-1)

N,M,P = np.shape(preds)

total_distance = 0

for i in range(N):

for j in range(M):

pred = preds[i][j][0]

true = trues[i][j][0]

min_value = min(pred, true)

max_value = max(pred, true)

total_distance += 1

if min_value >= 0:

total_distance -= (1 + min_value) / (1 + max_value)

else:

total_distance -= 1 / (1 + max_value + abs(min_value))

mean_distance = total_distance / (N*M)

return mean_distance

def r_squared(pred, true):

ssr = np.sum((pred - true) ** 2)

sst = np.sum((true - np.mean(true)) ** 2)

return 1 - (ssr/sst)

def metric(pred, true):

mae = MAE(pred, true)

mse = MSE(pred, true)

rmse = RMSE(pred, true)

mape = MAPE(pred, true)

mspe = MSPE(pred, true)

R2 = r_squared(pred, true)

mhd = MHD(pred, true)

rse = RSE(pred, true)

return mae, mse, rmse, mape, mspe, R2, mhd, rse

#------------------------------------------------------------metrics.py-------------------------------------------#

1.3.9 Generation of timefeatures.py

The code provides a collection of time feature classes that inherit from a base TimeFeature class, each converting different temporal components (seconds, minutes, hours, days, weeks, months) into normalized values between -0.5 and 0.5. The main functionality is exposed through two key functions: time_features_from_frequency_str() which returns appropriate time features based on a frequency string (like "12H", "5min", "1D"), and time_features() which converts a date index into a stacked array of these features.

To use this code, save it as 'timefeatures.py' and ensure you have pandas and numpy installed. The primary entry point is the time_features() function, which takes a pandas DatetimeIndex and an optional frequency parameter (default='h' for hourly). The code supports various time frequencies from yearly down to secondly granularity, making it particularly useful for time series modeling tasks where cyclical temporal patterns need to be encoded as numerical features.

The supported frequencies are: yearly (Y/A), monthly (M), weekly (W), daily (D), business days (B), hourly (H), minutely (T/min), and secondly (S). Each frequency automatically includes relevant time features at that level and above in the temporal hierarchy.

#---------------------------------------------------------timefeatures.py----------------------------------------#

from typing import List

import numpy as np

import pandas as pd

from pandas.tseries import offsets

from pandas.tseries.frequencies import to_offset

class TimeFeature:

def __init__(self):

pass

def __call__(self, index: pd.DatetimeIndex) -> np.ndarray:

pass

def __repr__(self):

return self.__class__.__name__ + "()"

class SecondOfMinute(TimeFeature):

"""Minute of hour encoded as value between [-0.5, 0.5]"""

def __call__(self, index: pd.DatetimeIndex) -> np.ndarray:

return index.second / 59.0 - 0.5

class MinuteOfHour(TimeFeature):

"""Minute of hour encoded as value between [-0.5, 0.5]"""

def __call__(self, index: pd.DatetimeIndex) -> np.ndarray:

return index.minute / 59.0 - 0.5

class HourOfDay(TimeFeature):

"""Hour of day encoded as value between [-0.5, 0.5]"""

def __call__(self, index: pd.DatetimeIndex) -> np.ndarray:

return index.hour / 23.0 - 0.5

class DayOfWeek(TimeFeature):

"""Hour of day encoded as value between [-0.5, 0.5]"""

def __call__(self, index: pd.DatetimeIndex) -> np.ndarray:

return index.dayofweek / 6.0 - 0.5

class DayOfMonth(TimeFeature):

"""Day of month encoded as value between [-0.5, 0.5]"""

def __call__(self, index: pd.DatetimeIndex) -> np.ndarray:

return (index.day - 1) / 30.0 - 0.5

class DayOfYear(TimeFeature):

"""Day of year encoded as value between [-0.5, 0.5]"""

def __call__(self, index: pd.DatetimeIndex) -> np.ndarray:

return (index.dayofyear - 1) / 365.0 - 0.5

class MonthOfYear(TimeFeature):

"""Month of year encoded as value between [-0.5, 0.5]"""

def __call__(self, index: pd.DatetimeIndex) -> np.ndarray:

return (index.month - 1) / 11.0 - 0.5

class WeekOfYear(TimeFeature):

"""Week of year encoded as value between [-0.5, 0.5]"""

def __call__(self, index: pd.DatetimeIndex) -> np.ndarray:

return (index.isocalendar().week - 1) / 52.0 - 0.5

def time_features_from_frequency_str(freq_str: str) -> List[TimeFeature]:

"""

Returns a list of time features that will be appropriate for the given frequency string.

Parameters

----------

freq_str

Frequency string of the form [multiple][granularity] such as "12H", "5min", "1D" etc.

"""

features_by_offsets = {

offsets.YearEnd: [],

offsets.QuarterEnd: [MonthOfYear],

offsets.MonthEnd: [MonthOfYear],

offsets.Week: [DayOfMonth, WeekOfYear],

offsets.Day: [DayOfWeek, DayOfMonth, DayOfYear],

offsets.BusinessDay: [DayOfWeek, DayOfMonth, DayOfYear],

offsets.Hour: [HourOfDay, DayOfWeek, DayOfMonth, DayOfYear],

offsets.Minute: [

MinuteOfHour,

HourOfDay,

DayOfWeek,

DayOfMonth,

DayOfYear,

],

offsets.Second: [

SecondOfMinute,

MinuteOfHour,

HourOfDay,

DayOfWeek,

DayOfMonth,

DayOfYear,

],

}

offset = to_offset(freq_str)

for offset_type, feature_classes in features_by_offsets.items():

if isinstance(offset, offset_type):

return [cls() for cls in feature_classes]

supported_freq_msg = f"""

Unsupported frequency {freq_str}

The following frequencies are supported:

Y - yearly

alias: A

M - monthly

W - weekly

D - daily

B - business days

H - hourly

T - minutely

alias: min

S - secondly

"""

raise RuntimeError(supported_freq_msg)

def time_features(dates, freq='h'):

return np.vstack([feat(dates) for feat in time_features_from_frequency_str(freq)])

#---------------------------------------------------------timefeatures.py----------------------------------------#

1.3.10 Generation of tools.py

The code contains several key utilities: a learning rate adjustment function (adjust_learning_rate) with two adjustment strategies, an early stopping mechanism (EarlyStopping) to prevent overfitting, a standard scaler implementation (StandardScaler) for data normalization, a dictionary extension (dotdict) that allows dot notation access, and a visualization function (visual) for plotting predictions against ground truth.

To use this code, save it as 'tools.py' and ensure you have PyTorch, NumPy, and Matplotlib installed. The learning rate adjustment supports two types of schedules: 'type1' (exponential decay) and 'type2' (predefined steps). The EarlyStopping class monitors validation loss and saves the best model checkpoint, while the StandardScaler handles data normalization and denormalization. The visualization function creates simple line plots comparing true values with predictions.

Most importantly, these tools are designed to work together in a deep learning training pipeline, with each component handling a specific aspect of the training process: learning rate scheduling, model checkpointing, data normalization, and results visualization. The visualization output is configured to use the 'agg' backend, making it suitable for environments without display capabilities.

#-------------------------------------------------------------tools.py---------------------------------------------#

import numpy as np

import torch

import matplotlib.pyplot as plt

plt.switch_backend('agg')

def adjust_learning_rate(optimizer, epoch, args):

# lr = args.learning_rate * (0.2 ** (epoch // 2))

if args.lradj == 'type1':

lr_adjust = {epoch: args.learning_rate * (0.5 ** ((epoch - 1) // 1))}

elif args.lradj == 'type2':

lr_adjust = {

2: 5e-5, 4: 1e-5, 6: 5e-6, 8: 1e-6,

10: 5e-7, 15: 1e-7, 20: 5e-8

}

if epoch in lr_adjust.keys():

lr = lr_adjust[epoch]

for param_group in optimizer.param_groups:

param_group['lr'] = lr

print('Updating learning rate to {}'.format(lr))

class EarlyStopping:

def __init__(self, patience=7, verbose=False, delta=0):

self.patience = patience

self.verbose = verbose

self.counter = 0

self.best_score = None

self.early_stop = False

self.val_loss_min = np.Inf

self.delta = delta

def __call__(self, val_loss, model, path):

score = -val_loss

if self.best_score is None:

self.best_score = score

self.save_checkpoint(val_loss, model, path)

elif score < self.best_score + self.delta:

self.counter += 1

print(f'EarlyStopping counter: {self.counter} out of {self.patience}')

if self.counter >= self.patience:

self.early_stop = True

else:

self.best_score = score

self.save_checkpoint(val_loss, model, path)

self.counter = 0

def save_checkpoint(self, val_loss, model, path):

if self.verbose:

print(f'Validation loss decreased ({self.val_loss_min:.6f} --> {val_loss:.6f}). Saving model ...')

torch.save(model.state_dict(), path + '/' + 'checkpoint.pth')

self.val_loss_min = val_loss

class dotdict(dict):

"""dot.notation access to dictionary attributes"""

__getattr__ = dict.get

__setattr__ = dict.__setitem__

__delattr__ = dict.__delitem__

class StandardScaler():

def __init__(self, mean, std):

self.mean = mean

self.std = std

def transform(self, data):

return (data - self.mean) / self.std

def inverse_transform(self, data):

return (data * self.std) + self.mean

def visual(true, preds=None, name='./pic/test.pdf'):

"""

Results visualization

"""

plt.figure()

plt.plot(true, label='GroundTruth', linewidth=2)

if preds is not None:

plt.plot(preds, label='Prediction', linewidth=2)

plt.legend()

plt.savefig(name, bbox_inches='tight')

#-------------------------------------------------------------tools.py---------------------------------------------#

1.3.11 Generation of CNNCorrelationBasedTransformer.py

This code implements a CNN Correlation-based Transformer model designed for time series forecasting. The model builds upon the traditional Transformer architecture but introduces a novel correlation mechanism that captures periodic dependencies in time series data. The implementation consists of 17 main components, including 16 classes and 1 utility function:

1. version_inspection (utility function for version comparison)
2. Model (main class)
3. my_Layernorm
4. moving_avg
5. SeriesDecomposing
6. EncoderLayer
7. Encoder
8. DecoderLayer
9. Decoder
10. Correlation (key innovation)
11. CorrelationLayer
12. TokenEmbedding
13. FixedEncoding
14. TemporalEncoding
15. TimeFeatureEncoding
16. DataEncoding

The key innovation lies in the Correlation class, which replaces traditional attention mechanisms with a CNN correlation-based approach. It uses Fast Fourier Transform (FFT) to identify periodic patterns and implements a Time Shift and Concatenation (TSC) mechanism that aggregates information across different time shifts. The version_inspection function is a utility that compares version numbers (like "1.5.0" vs "1.4.0") to ensure compatibility with different PyTorch versions, particularly used in the TokenEmbedding class for padding calculations.

To reproduce this model:

1. Ensure you have PyTorch installed with FFT support.
2. The model requires configuration parameters including sequence length (seq_len), label length (label_len), prediction length (pred_len), and model dimensions (d_model).
3. Input data should include both the time series values and temporal markers (like hour, day, month).
4. The model processes data through an encoder-decoder architecture, with CNN correlation-based attention mechanisms in both components.

Developers—including users, scholars, or researchers may copy the given code and paste it in a file CNNCorrelationBasedTransformer.py and save it.

#-----------------------------------------CNNCorrelationBasedTransformer.py--------------------------------#

import torch

import torch.nn as nn

import torch.nn.functional as F

import numpy as np

import matplotlib.pyplot as plt

import math

from math import sqrt

import os

from torch.nn.utils import weight_norm

def version_inspection(ver1, ver2):

"""

:param ver1

:param ver2

:return: ver1< = >ver2 False/True

"""

list1 = str(ver1).split(".")

list2 = str(ver2).split(".")

for i in range(len(list1)) if len(list1) < len(list2) else range(len(list2)):

if int(list1[i]) == int(list2[i]):

pass

elif int(list1[i]) < int(list2[i]):

return -1

else:

return 1

if len(list1) == len(list2):

return True

elif len(list1) < len(list2):

return False

else:

return True

class Model(nn.Module):

def __init__(self, configs):

super(Model, self).__init__()

self.seq_len = configs.seq_len

self.label_len = configs.label_len

self.pred_len = configs.pred_len

self.output_attention = configs.output_attention

# Series Decomposing

kernel_size = configs.moving_avg

self.decomp = SeriesDecomposing(kernel_size)

# Embedding

# The series-wise connection inherently contains the sequential information.

# Thus, we can discard the position embedding of transformers.

self.enc_embedding = DataEmbedding(configs.enc_in, configs.d_model, configs.embed, configs.freq,

configs.dropout)

self.dec_embedding = DataEmbedding(configs.dec_in, configs.d_model, configs.embed, configs.freq,

configs.dropout)

# Encoder

self.encoder = Encoder(

[

EncoderLayer(

CorrelationLayer(

Correlation(False, configs.factor, attention_dropout=configs.dropout,

output_attention=configs.output_attention),

configs.d_model, configs.cnn_out_channels, configs.n_heads),

configs.d_model,

configs.d_ff,

moving_avg=configs.moving_avg,

dropout=configs.dropout,

activation=configs.activation

) for l in range(configs.e_layers)

],

norm_layer=my_Layernorm(configs.d_model)

)

# Decoder

self.decoder = Decoder(

[

DecoderLayer(

CorrelationLayer(

Correlation(True, configs.factor, attention_dropout=configs.dropout,

output_attention=False),

configs.d_model, configs.cnn_out_channels, configs.n_heads),

CorrelationLayer(

Correlation(False, configs.factor, attention_dropout=configs.dropout,

output_attention=False),

configs.d_model, configs.cnn_out_channels, configs.n_heads),

configs.d_model,

configs.c_out,

configs.d_ff,

moving_avg=configs.moving_avg,

dropout=configs.dropout,

activation=configs.activation,

)

for l in range(configs.d_layers)

],

norm_layer=my_Layernorm(configs.d_model),

projection=nn.Linear(configs.d_model, configs.c_out, bias=True)

)

def forward(self, x_enc, x_mark_enc, x_dec, x_mark_dec,

enc_self_mask=None, dec_self_mask=None, dec_enc_mask=None):

# decomp init

mean = torch.mean(x_enc, dim=1).unsqueeze(1).repeat(1, self.pred_len, 1)

zeros = torch.zeros([x_dec.shape[0], self.pred_len, x_dec.shape[2]], device=x_enc.device)

seasonal_init, trend_init = self.decomp(x_enc)

# decoder input

trend_init = torch.cat([trend_init[:, -self.label_len:, :], mean], dim=1)

seasonal_init = torch.cat([seasonal_init[:, -self.label_len:, :], zeros], dim=1)

# enc

enc_out = self.enc_embedding(x_enc, x_mark_enc)

enc_out, attns = self.encoder(enc_out, attn_mask=enc_self_mask)

# dec

dec_out = self.dec_embedding(seasonal_init, x_mark_dec)

seasonal_part, trend_part = self.decoder(dec_out, enc_out, x_mask=dec_self_mask, cross_mask=dec_enc_mask,

trend=trend_init)

# final

dec_out = trend_part + seasonal_part

if self.output_attention:

return dec_out[:, -self.pred_len:, :], attns

else:

return dec_out[:, -self.pred_len:, :] # [B, L, D]

class my_Layernorm(nn.Module):

"""

Special designed layernorm for the seasonal part

"""

def __init__(self, channels):

super(my_Layernorm, self).__init__()

self.layernorm = nn.LayerNorm(channels)

def forward(self, x):

x_hat = self.layernorm(x)

bias = torch.mean(x_hat, dim=1).unsqueeze(1).repeat(1, x.shape[1], 1)

return x_hat - bias

class moving_avg(nn.Module):

def __init__(self, kernel_size, stride):

super(moving_avg, self).__init__()

self.kernel_size = kernel_size

self.avg = nn.AvgPool1d(kernel_size=kernel_size, stride=stride, padding=0)

def forward(self, x):

# padding on the both ends of time series

front = x[:, 0:1, :].repeat(1, (self.kernel_size - 1) // 2, 1)

end = x[:, -1:, :].repeat(1, (self.kernel_size - 1) // 2, 1)

x = torch.cat([front, x, end], dim=1)

x = self.avg(x.permute(0, 2, 1))

x = x.permute(0, 2, 1)

return x

class SeriesDecomposing(nn.Module):

def __init__(self, kernel_size):

super(SeriesDecomposing, self).__init__()

self.moving_avg = moving_avg(kernel_size, stride=1)

def forward(self, x):

moving_mean = self.moving_avg(x)

res = x - moving_mean

return res, moving_mean

class EncoderLayer(nn.Module):

def __init__(self, attention, d_model, d_ff=None, moving_avg=25, dropout=0.1, activation="relu"):

super(EncoderLayer, self).__init__()

d_ff = d_ff or 4 * d_model

self.attention = attention

self.conv1 = nn.Conv1d(in_channels=d_model, out_channels=d_ff, kernel_size=1, bias=False)

self.conv2 = nn.Conv1d(in_channels=d_ff, out_channels=d_model, kernel_size=1, bias=False)

self.decomp1 = SeriesDecomposing(moving_avg)

self.decomp2 = SeriesDecomposing(moving_avg)

self.dropout = nn.Dropout(dropout)

self.activation = F.relu if activation == "relu" else F.gelu

def forward(self, x, attn_mask=None):

new_x, attn = self.attention(

x, x, x,

attn_mask=attn_mask

)

x = x + self.dropout(new_x)

x, _ = self.decomp1(x)

y = x

y = self.dropout(self.activation(self.conv1(y.transpose(-1, 1))))

y = self.dropout(self.conv2(y).transpose(-1, 1))

res, _ = self.decomp2(x + y)

return res, attn

class Encoder(nn.Module):

def __init__(self, attn_layers, conv_layers=None, norm_layer=None):

super(Encoder, self).__init__()

self.attn_layers = nn.ModuleList(attn_layers)

self.conv_layers = nn.ModuleList(conv_layers) if conv_layers is not None else None

self.norm = norm_layer

def forward(self, x, attn_mask=None):

attns = []

if self.conv_layers is not None:

for attn_layer, conv_layer in zip(self.attn_layers, self.conv_layers):

x, attn = attn_layer(x, attn_mask=attn_mask)

x = conv_layer(x)

attns.append(attn)

x, attn = self.attn_layers[-1](x)

attns.append(attn)

else:

for attn_layer in self.attn_layers:

x, attn = attn_layer(x, attn_mask=attn_mask)

attns.append(attn)

if self.norm is not None:

x = self.norm(x)

return x, attns

class DecoderLayer(nn.Module):

def __init__(self, self_attention, cross_attention, d_model, c_out, d_ff=None,

moving_avg=25, dropout=0.1, activation="relu"):

super(DecoderLayer, self).__init__()

d_ff = d_ff or 4 * d_model

self.self_attention = self_attention

self.cross_attention = cross_attention

self.conv1 = nn.Conv1d(in_channels=d_model, out_channels=d_ff, kernel_size=1, bias=False)

self.conv2 = nn.Conv1d(in_channels=d_ff, out_channels=d_model, kernel_size=1, bias=False)

self.decomp1 = SeriesDecomposing(moving_avg)

self.decomp2 = SeriesDecomposing(moving_avg)

self.decomp3 = SeriesDecomposing(moving_avg)

self.dropout = nn.Dropout(dropout)

self.projection = nn.Conv1d(in_channels=d_model, out_channels=c_out, kernel_size=3, stride=1, padding=1,

padding_mode='circular', bias=False)

self.activation = F.relu if activation == "relu" else F.gelu

def forward(self, x, cross, x_mask=None, cross_mask=None):

x = x + self.dropout(self.self_attention(

x, x, x,

attn_mask=x_mask

)[0])

x, trend1 = self.decomp1(x)

x = x + self.dropout(self.cross_attention(

x, cross, cross,

attn_mask=cross_mask

)[0])

x, trend2 = self.decomp2(x)

y = x

y = self.dropout(self.activation(self.conv1(y.transpose(-1, 1))))

y = self.dropout(self.conv2(y).transpose(-1, 1))

x, trend3 = self.decomp3(x + y)

residual_trend = trend1 + trend2 + trend3

residual_trend = self.projection(residual_trend.permute(0, 2, 1)).transpose(1, 2)

return x, residual_trend

class Decoder(nn.Module):

def __init__(self, layers, norm_layer=None, projection=None):

super(Decoder, self).__init__()

self.layers = nn.ModuleList(layers)

self.norm = norm_layer

self.projection = projection

def forward(self, x, cross, x_mask=None, cross_mask=None, trend=None):

for layer in self.layers:

x, residual_trend = layer(x, cross, x_mask=x_mask, cross_mask=cross_mask)

trend = trend + residual_trend

if self.norm is not None:

x = self.norm(x)

if self.projection is not None:

x = self.projection(x)

return x, trend

class Correlation(nn.Module):

def __init__(self, mask_flag=True, factor=1, scale=None, attention_dropout=0.1, output_attention=False):

super(Correlation, self).__init__()

self.factor = factor

self.scale = scale

self.mask_flag = mask_flag

self.output_attention = output_attention

self.dropout = nn.Dropout(attention_dropout)

#TSC stands for Time Shift and Concatenation

def TSC_training(self, values, corr):

head = values.shape[1]

channel = values.shape[2]

length = values.shape[3]

# find top k

top_k = int(self.factor * math.log(length))

mean_value = torch.mean(torch.mean(corr, dim=1), dim=1)

index = torch.topk(torch.mean(mean_value, dim=0), top_k, dim=-1)[1]

weights = torch.stack([mean_value[:, index[i]] for i in range(top_k)], dim=-1)

# update corr

tmp_corr = torch.softmax(weights, dim=-1)

# aggregation

tmp_values = values

delays_agg = torch.zeros_like(values).float()

for i in range(top_k):

pattern = torch.roll(tmp_values, -int(index[i]), -1)

delays_agg = delays_agg + pattern * \

(tmp_corr[:, i].unsqueeze(1).unsqueeze(1).unsqueeze(1).repeat(1, head, channel, length))

return delays_agg

def TSC_inference(self, values, corr):

batch = values.shape[0]

head = values.shape[1]

channel = values.shape[2]

length = values.shape[3]

# index init

init_index = torch.arange(length).unsqueeze(0).unsqueeze(0).unsqueeze(0)\

.repeat(batch, head, channel, 1).to(values.device)

# find top k

top_k = int(self.factor * math.log(length))

mean_value = torch.mean(torch.mean(corr, dim=1), dim=1)

weights, delay = torch.topk(mean_value, top_k, dim=-1)

# update corr

tmp_corr = torch.softmax(weights, dim=-1)

# aggregation

tmp_values = values.repeat(1, 1, 1, 2)

delays_agg = torch.zeros_like(values).float()

for i in range(top_k):

tmp_delay = init_index + delay[:, i].unsqueeze(1).unsqueeze(1).unsqueeze(1).repeat(1, head, channel, length)

pattern = torch.gather(tmp_values, dim=-1, index=tmp_delay)

delays_agg = delays_agg + pattern * \

(tmp_corr[:, i].unsqueeze(1).unsqueeze(1).unsqueeze(1).repeat(1, head, channel, length))

return delays_agg

def TSC_full(self, values, corr):

batch = values.shape[0]

head = values.shape[1]

channel = values.shape[2]

length = values.shape[3]

# index init

init_index = torch.arange(length).unsqueeze(0).unsqueeze(0).unsqueeze(0)\

.repeat(batch, head, channel, 1).to(values.device)

# find top k

top_k = int(self.factor * math.log(length))

weights, delay = torch.topk(corr, top_k, dim=-1)

# update corr

tmp_corr = torch.softmax(weights, dim=-1)

# aggregation

tmp_values = values.repeat(1, 1, 1, 2)

delays_agg = torch.zeros_like(values).float()

for i in range(top_k):

tmp_delay = init_index + delay[..., i].unsqueeze(-1)

pattern = torch.gather(tmp_values, dim=-1, index=tmp_delay)

delays_agg = delays_agg + pattern * (tmp_corr[..., i].unsqueeze(-1))

return delays_agg

def forward(self, queries, keys, values, attn_mask):

B, L, H, E = queries.shape

_, S, _, D = values.shape

if L > S:

zeros = torch.zeros_like(queries[:, :(L - S), :]).float()

values = torch.cat([values, zeros], dim=1)

keys = torch.cat([keys, zeros], dim=1)

else:

values = values[:, :L, :, :]

keys = keys[:, :L, :, :]

# period-based dependencies

q_fft = torch.fft.rfft(queries.permute(0, 2, 3, 1).contiguous(), dim=-1)

k_fft = torch.fft.rfft(keys.permute(0, 2, 3, 1).contiguous(), dim=-1)

res = q_fft * torch.conj(k_fft)

corr = torch.fft.irfft(res, n=L, dim=-1)

# time delay agg

if self.training:

V = self.TSC_training(values.permute(0, 2, 3, 1).contiguous(), corr).permute(0, 3, 1, 2)

else:

V = self.TSC_inference(values.permute(0, 2, 3, 1).contiguous(), corr).permute(0, 3, 1, 2)

if self.output_attention:

return (V.contiguous(), corr.permute(0, 3, 1, 2))

else:

return (V.contiguous(), None)

class CorrelationLayer(nn.Module):

def __init__(self, correlation, d_model, n_heads, d_keys=None,

d_values=None, cnn_out_channels=128, kernel_size=1):

super(CorrelationLayer, self).__init__()

d_keys = d_keys or (d_model // n_heads)

d_values = d_values or (d_model // n_heads)

self.inner_correlation = correlation

#Convert time-series data to CNN information for queries, keys, and values

self.cnn_queries = nn.Conv1d(in_channels=d_model, out_channels=cnn_out_channels, kernel_size=kernel_size, padding=kernel_size // 2)

self.cnn_keys = nn.Conv1d(in_channels=d_model, out_channels=cnn_out_channels, kernel_size=kernel_size, padding=kernel_size // 2)

self.cnn_values = nn.Conv1d(in_channels=d_model, out_channels=cnn_out_channels, kernel_size=kernel_size, padding=kernel_size // 2)

#Change projection layers to align with the input size of each CNN information

self.query_projection = nn.Linear(cnn_out_channels, d_keys * n_heads)

self.key_projection = nn.Linear(cnn_out_channels, d_keys * n_heads)

self.value_projection = nn.Linear(cnn_out_channels, d_values * n_heads)

#

self.out_projection = nn.Linear(d_values * n_heads, d_model)

self.n_heads = n_heads

def forward(self, queries, keys, values, attn_mask):

B, L, _ = queries.shape

_, S, _ = keys.shape

H = self.n_heads

#Perform input transposing to align with CNN attributions namely, batch, channels, length

queries = queries.transpose(1, 2)

keys = keys.transpose(1, 2)

values = values.transpose(1, 2)

queries = self.cnn_queries(queries).transpose(1, 2)

keys = self.cnn_keys(keys).transpose(1, 2)

values = self.cnn_values(values).transpose(1, 2)

queries = self.query_projection(queries).view(B, L, H, -1)

keys = self.key_projection(keys).view(B, S, H, -1)

values = self.value_projection(values).view(B, S, H, -1)

#

out, attn = self.inner_correlation(

queries,

keys,

values,

attn_mask

)

out = out.view(B, L, -1)

return self.out_projection(out), attn

class TokenEmbedding(nn.Module):

def __init__(self, c_in, d_model):

super(TokenEmbedding, self).__init__()

padding = 1 if version_inspection(torch.__version__, '1.5.0') else 2

self.tokenConv = nn.Conv1d(in_channels=c_in, out_channels=d_model,

kernel_size=3, padding=padding, padding_mode='circular', bias=False)

for m in self.modules():

if isinstance(m, nn.Conv1d):

nn.init.kaiming_normal_(m.weight, mode='fan_in', nonlinearity='leaky_relu')

def forward(self, x):

x = self.tokenConv(x.permute(0, 2, 1)).transpose(1, 2)

return x

class FixedEmbedding(nn.Module):

def __init__(self, c_in, d_model):

super(FixedEmbedding, self).__init__()

w = torch.zeros(c_in, d_model).float()

w.require_grad = False

position = torch.arange(0, c_in).float().unsqueeze(1)

div_term = (torch.arange(0, d_model, 2).float() * -(math.log(10000.0) / d_model)).exp()

w[:, 0::2] = torch.sin(position * div_term)

w[:, 1::2] = torch.cos(position * div_term)

self.emb = nn.Embedding(c_in, d_model)

self.emb.weight = nn.Parameter(w, requires_grad=False)

def forward(self, x):

return self.emb(x).detach()

class TemporalEmbedding(nn.Module):

def __init__(self, d_model, embed_type='fixed', freq='h'):

super(TemporalEmbedding, self).__init__()

minute_size = 4

hour_size = 24

weekday_size = 7

day_size = 32

month_size = 13

Embed = FixedEmbedding if embed_type == 'fixed' else nn.Embedding

if freq == 't':

self.minute_embed = Embed(minute_size, d_model)

self.hour_embed = Embed(hour_size, d_model)

self.weekday_embed = Embed(weekday_size, d_model)

self.day_embed = Embed(day_size, d_model)

self.month_embed = Embed(month_size, d_model)

def forward(self, x):

x = x.long()

minute_x = self.minute_embed(x[:, :, 4]) if hasattr(self, 'minute_embed') else 0.

hour_x = self.hour_embed(x[:, :, 3])

weekday_x = self.weekday_embed(x[:, :, 2])

day_x = self.day_embed(x[:, :, 1])

month_x = self.month_embed(x[:, :, 0])

return hour_x + weekday_x + day_x + month_x + minute_x

class TimeFeatureEmbedding(nn.Module):

def __init__(self, d_model, embed_type='timeF', freq='h'):

super(TimeFeatureEmbedding, self).__init__()

freq_map = {'h': 4, 't': 5, 's': 6, 'm': 1, 'a': 1, 'w': 2, 'd': 3, 'b': 3}

d_inp = freq_map[freq]

self.embed = nn.Linear(d_inp, d_model, bias=False)

def forward(self, x):

return self.embed(x)

class DataEmbedding(nn.Module):

def __init__(self, c_in, d_model, embed_type='fixed', freq='h', dropout=0.1):

super(DataEmbedding, self).__init__()

self.value_embedding = TokenEmbedding(c_in=c_in, d_model=d_model)

self.temporal_embedding = TemporalEmbedding(d_model=d_model, embed_type=embed_type,

freq=freq) if embed_type != 'timeF' else TimeFeatureEmbedding(

d_model=d_model, embed_type=embed_type, freq=freq)

self.dropout = nn.Dropout(p=dropout)

def forward(self, x, x_mark):

x = self.value_embedding(x) + self.temporal_embedding(x_mark)

return self.dropout(x)

#-----------------------------------------CNNCorrelationBasedTransformer.py--------------------------------#

1.3.12 Generation of Execute.ipynb

This code implements a Time Series Forecasting system using a CNN Correlation-based Transformer model. The code is structured in two main parts that should be copied into separate cells in your Jupyter notebook:

Cell [1]: Contains declarations.

Cell [2]: Contains the necessary imports including PyTorch, NumPy, and custom utilities. This section sets up the basic framework for the implementation.

Cell [3]: Contains the complete configuration setup, including argument parsing and parameter initialization. It defines all hyperparameters for the model architecture, data loading, training process, and GPU usage. The code uses argparse to create a flexible command-line interface, though parameters are pre-set in the script.

Key Points about Transfer Learning Implementation:

1. The code includes commented-out sections (marked with #) that implement transfer learning.
2. To activate transfer learning:
   - Uncomment the lines starting with #import CNNCorrelationBasedTransformer.
   - Uncomment the model loading section to load a pre-trained model from checkpoints.
   - Uncomment the encoder freezing section to prevent updates to the encoder parameters.

Storage Organization:

- Checkpoints folder (./checkpoints/): Stores model parameters and configuration settings in .pth files.
- Results folder (./results/): Contains the output metrics, predictions, and performance evaluations.

To reproduce:

1. Create a new Jupyter notebook.
2. Copy everything from the import section (Cell [1]) into your first cell.
3. Copy the entire configuration and execution code (Cell [2]) into your second cell.
4. Ensure you have the required data files in the specified ./data/ directory.
5. Save the Jupyter notebook as **“Execute.ipynb.”**
6. Run the cells in order.

The model will automatically handle training, testing, and prediction phases. Training configurations and model states will be saved in the checkpoints folder, while all performance metrics and prediction results will be stored in the results folder for further analysis.

#--------------------------------------------------------Execute.ipynb-------------------------------------------#

Cell [1]

#The original code developed by Haixu Wu, available on GitHub, was adapted by the authors, with Ling Feng leading the adaptation, to meet the requirements of the experiments.

#All modifications to the original code were made in accordance with the MIT License below:

#MIT License

#Copyright (c) 2021 THUML @ Tsinghua University

#Permission is hereby granted, free of charge, to any person obtaining a copy of this software and associated

#documentation files (the \"Software\"), to deal in the Software without restriction, including without limitation

#the rights to use, copy, modify, merge, publish, distribute, sublicense, and/or sell copies of the Software,

#and to permit persons to whom the Software is furnished to do so, subject to the following conditions:

#The above copyright notice and this permission notice shall be included in all copies or substantial portions of the Software

#THE SOFTWARE IS PROVIDED \"AS IS\", WITHOUT WARRANTY OF ANY KIND, EXPRESS OR IMPLIED, INCLUDING BUT NOT LIMITED TO

#THE WARRANTIES OF MERCHANTABILITY, FITNESS FOR A PARTICULAR PURPOSE AND NONINFRINGEMENT. IN NO EVENT SHALL THE AUTHORS

#OR COPYRIGHT HOLDERS BE LIABLE FOR ANY CLAIM, DAMAGES OR OTHER LIABILITY, WHETHER IN AN ACTION OF CONTRACT, TORT OR

#OTHERWISE, ARISING FROM, OUT OF OR IN CONNECTION WITH THE SOFTWARE OR THE USE OR OTHER DEALINGS IN THE SOFTWARE."

Cell [2]

import argparse

import random

import os

import matplotlib.pyplot as plt

import numpy as np

import torch

from utils.tools import dotdict

from exp.exp_main import Exp_Main

import sys

Cell [3]

fix_seed = 2021

random.seed(fix_seed)

torch.manual_seed(fix_seed)

np.random.seed(fix_seed)

parser = argparse.ArgumentParser(description='Time Series Forecasting Using CNN Correlation-based Transformer')

# basic config

parser.add_argument('--is_training', type=int, required=True, default=1, help='status')

parser.add_argument('--model_id', type=str, required=True, default='test', help='model id')

parser.add_argument('--model', type=str, required=True, default='CNNCorrelationBasedTransformer',

help='model name, there is no option for this, only CNNCorrelationBasedTransformer available')

# data loader

parser.add_argument('--data', type=str, required=True, default='custom', help='dataset type')

parser.add_argument('--root_path', type=str, default='./data/', help='root path of the data file')

parser.add_argument('--data_path', type=str, default='set2024.csv', help='data file')

parser.add_argument('--features', type=str, default='MS',

help='forecasting task, options:[M, S, MS]; M:multivariate predict multivariate, S:univariate predict univariate, MS:multivariate predict univariate')

parser.add_argument('--target', type=str, default='OT', help='target feature in S or MS task')

parser.add_argument('--freq', type=str, default='d',

help='freq for time features encoding, options:[s:secondly, t:minutely, h:hourly, d:daily, b:business days, w:weekly, m:monthly], you can also use more detailed freq like 15min or 3h')

parser.add_argument('--checkpoints', type=str, default='./checkpoints/', help='location of model checkpoints')

# forecasting task

parser.add_argument('--seq_len', type=int, default=10, help='input sequence length')

parser.add_argument('--label_len', type=int, default=10, help='start token length')

parser.add_argument('--pred_len', type=int, default=5, help='prediction sequence length')

# model define

parser.add_argument('--enc_in', type=int, default=4, help='encoder input size')

parser.add_argument('--dec_in', type=int, default=4, help='decoder input size')

parser.add_argument('--c_out', type=int, default=1, help='output size')

parser.add_argument('--cnn_out_channels', type=int, default=512, help='dimension of CNN')

parser.add_argument('--d_model', type=int, default=512, help='dimension of model')

parser.add_argument('--n_heads', type=int, default=4, help='num of heads')

parser.add_argument('--e_layers', type=int, default=2, help='num of encoder layers')

parser.add_argument('--d_layers', type=int, default=1, help='num of decoder layers')

parser.add_argument('--d_ff', type=int, default=2048, help='dimension of fcn')

parser.add_argument('--moving_avg', type=int, default=35, help='window size of moving average')

parser.add_argument('--factor', type=int, default=4, help='attn factor')

parser.add_argument('--distil', action='store_false',

help='whether to use distilling in encoder, using this argument means not using distilling',

default=True)

parser.add_argument('--dropout', type=float, default=0.05, help='dropout')

parser.add_argument('--embed', type=str, default='timeF',

help='time features encoding, options:[timeF, fixed, learned]')

parser.add_argument('--activation', type=str, default='gelu', help='activation')

parser.add_argument('--output_attention', action='store_true', help='whether to output attention in encoder')

parser.add_argument('--do_predict', action='store_true', help='whether to predict unseen future data')

# optimization

parser.add_argument('--num_workers', type=int, default=0, help='data loader num workers')

parser.add_argument('--itr', type=int, default=2, help='experiments times')

parser.add_argument('--train_epochs', type=int, default=30, help='train epochs')

parser.add_argument('--batch_size', type=int, default=8, help='batch size of train input data')

parser.add_argument('--patience', type=int, default=20, help='early stopping patience')

parser.add_argument('--learning_rate', type=float, default=0.0001, help='optimizer learning rate')

parser.add_argument('--des', type=str, default='test', help='exp description')

parser.add_argument('--loss', type=str, default='mse', help='loss function')

parser.add_argument('--lradj', type=str, default='type1', help='adjust learning rate')

parser.add_argument('--use_amp', action='store_true', help='use automatic mixed precision training', default=False)

# GPU

parser.add_argument('--use_gpu', type=bool, default=True, help='use gpu')

parser.add_argument('--gpu', type=int, default=0, help='gpu')

parser.add_argument('--use_multi_gpu', action='store_true', help='use multiple gpus', default=False)

parser.add_argument('--devices', type=str, default='0,1,2,3', help='device ids of multile gpus')

sys.argv = ['TSF Using CBT', '--is_training', '1', '--model_id', 'test', '--model', 'CNNCorrelationBasedTransformer',

'--data', 'custom',

'--root_path', './data/',

'--data_path', 'set2024.csv',

'--features', 'MS',

'--target', 'OT',

'--freq', 'd',

'--checkpoints', './checkpoints/',

'--seq_len', '10',

'--label_len', '10',

'--pred_len', '5',

'--enc_in', '4',

'--dec_in', '4',

'--c_out', '1',

'--cnn_out_channels', '128',

'--d_model', '512',

'--n_heads', '4',

'--e_layers', '2',

'--d_layers', '1',

'--d_ff', '2048',

'--moving_avg', '35',

'--factor', '4',

'--distil',

'--dropout', '0.05',

'--embed', 'timeF',

'--activation', 'gelu',

'--output_attention',

'--do_predict',

'--num_workers', '0',

'--itr', '2',

'--train_epochs', '30',

'--batch_size', '8',

'--patience', '20',

'--learning_rate', '0.0001',

'--des', 'test',

'--loss', 'mse',

'--lradj', 'type1',

'--use_amp',

'--use_gpu', False, #True,

'--gpu', '0',

'--use_multi_gpu',

'--devices', '0'

]

args = parser.parse_args()

args.use_gpu = True if torch.cuda.is_available() and args.use_gpu else False

if args.use_gpu and args.use_multi_gpu:

args.dvices = args.devices.replace(' ', '')

device_ids = args.devices.split(',')

args.device_ids = [int(id_) for id_ in device_ids]

args.gpu = args.device_ids[0]

print('Args in experiment:')

print(args)

### This is where the proposed method of transfer learning takes place. ###

### Once the model is trained/validated/tested to satisfy the requirements, ###

### just remove the symbol "#" in the following lines as indicated below ###

### to perform transfer learning. ###

### The parameter settings of each training/validating/testing are saved in a file ended with ".pth" ###

### in the "checkpoints" folder. Also, the filename is defined the parser section up above. ###

#import CNNCorrelationBasedTransformer ## Remove the pound sign at front ##

# Load pre-trained model

#model = CNNCorrelationBasedTransformer.Model(args) ## Remove the pound sign at front ##

#checkpoint_path = './checkpoints/**---Folder of Trained Parameter---/**checkpoint.pth' ## Remove the pound sign at front ##

#model.load_state_dict(torch.load(checkpoint_path)) ## Remove the pound sign at front ##

# Freeze encoder layers

#for param in model.encoder.parameters(): ## Remove the pound sign at front ##

# param.requires_grad = False ## Remove the pound sign at front ##

#for name, param in model.named_parameters(): ## Remove the pound sign at front ##

# if 'EncoderLayer.0' in name: ## Remove the pound sign at front ##

# param.requires_grad = False ## Remove the pound sign at front ##

Exp = Exp_Main

if args.is_training:

for ii in range(args.itr):

# setting record of experiments

setting = '{}_{}_{}_ft{}_sl{}_ll{}_pl{}_dcc{}_dm{}_nh{}_el{}_dl{}_df{}_fc{}_eb{}_dt{}_{}_{}'.format(

args.model_id,

args.model,

args.data,

args.features,

args.seq_len,

args.label_len,

args.pred_len,

args.cnn_out_channels,

args.d_model,

args.n_heads,

args.e_layers,

args.d_layers,

args.d_ff,

args.factor,

args.embed,

args.distil,

args.des, ii)

exp = Exp(args) # set experiments

print('>>>>>>>start training : {}>>>>>>>>>>>>>>>>>>>>>>>>>>'.format(setting))

exp.train(setting)

print('>>>>>>>testing : {}<<<<<<<<<<<<<<<<<<<<<<<<<<<<<<<<<'.format(setting))

exp.test(setting)

if args.do_predict:

print('>>>>>>>predicting : {}<<<<<<<<<<<<<<<<<<<<<<<<<<<<<<<<<'.format(setting))

exp.predict(setting, True)

torch.cuda.empty_cache()

else:

ii = 0

setting = '{}_{}_{}_ft{}_sl{}_ll{}_pl{}_dcc{}_dm{}_nh{}_el{}_dl{}_df{}_fc{}_eb{}_dt{}_{}_{}'.format(

args.model_id,

args.model,

args.data,

args.features,

args.seq_len,

args.label_len,

args.pred_len,

args.cnn_out_channels,

args.d_model,

args.n_heads,

args.e_layers,

args.d_layers,

args.d_ff,

args.factor,

args.embed,

args.distil,

args.des, ii)

exp = Exp(args) # set experiments

print('>>>>>>>testing : {}<<<<<<<<<<<<<<<<<<<<<<<<<<<<<<<<<'.format(setting))

exp.test(setting, test=1)

torch.cuda.empty_cache()

#--------------------------------------------------------Execute.ipynb-------------------------------------------#

**2. IMPLEMENTATION AND EXECUTION OF THE PROPOSED METHOD**

The proposed transfer learning method consists of two main phases: source domain training and target domain adaptation.

First, in the source domain phase:

1. Input data is fed through both encoder and decoder.
2. After training, validation, and testing, the model parameters are saved as a pre-trained model (.pth file) in the "checkpoints" directory.

Then, for the target domain phase:

1. Load the pre-trained model.
2. Freeze the encoder parameters while keeping the decoder parameters trainable.
3. Process target domain data through both encoder and decoder.
4. Train, validate, and test the model until desired performance is achieved.
5. Save the final target domain model parameters.

To make predictions using either model:

1. Load the corresponding model parameter file.
2. Set the --is_training parameter to "0" in sys.argv within "Execute.ipynb."
3. To utilize GPU acceleration, set the --use_gpu parameter to "True."
4. Run "Execute.ipynb."
